# A novel rat model of comorbid PTSD and addiction reveals intersections between stress susceptibility and enhanced cocaine seeking with a role for mGlu5 receptors

**DOI:** 10.1038/s41398-018-0265-9

**Published:** 2018-10-05

**Authors:** Marek Schwendt, John Shallcross, Natalie A. Hadad, Mark D. Namba, Helmut Hiller, Lizhen Wu, Eric G. Krause, Lori A. Knackstedt

**Affiliations:** 1 Psychology Department, University of Florida, Gainesville, FL 32611 USA; 2 Center for Addiction Research and Education, University of Florida, Gainesville, FL 32610 USA; 3 Department of Pharmacodynamics, University of Florida, Gainesville, FL 32610 USA

## Abstract

PTSD is highly comorbid with cocaine use disorder (CUD), and cocaine users with PTSD + CUD are more resistant to treatment. Here we sought to develop a rat model of PTSD + CUD in order to identify the neurobiological changes underlying such comorbidity and screen potential medications for reducing cocaine seeking in the PTSD population. We utilized a predator scent stress model of PTSD, wherein rats received a single exposure to the fox pheromone 2,5-dihydro-2,4,5-trimethylthiazoline (TMT). One week after TMT exposure, stress-susceptible (susceptible), intermediate, and resilient phenotypes were detected and were consistent with behavioral, corticosterone, and gene expression profiles 3 weeks post TMT. We assessed phenotypic differences in cocaine self-administration, extinction, and cue-primed reinstatement. Susceptible rats exhibited deficits in extinction learning and increased cue-primed reinstatement that was not prevented by Ceftriaxone, an antibiotic that consistently attenuates the reinstatement of cocaine seeking. TMT-exposed resilient rats displayed increased *mGlu5* gene expression in the amygdala and medial prefrontal cortex and did not display the enhanced cocaine seeking observed in susceptible rats. Combined treatment with the mGlu5 positive allosteric modulator 3-Cyano-N-(1,3-diphenyl-1 H-pyrazol-5-yl)benzamide (CDPPB), fear extinction, and ceftriaxone prevented the reinstatement of cocaine seeking in susceptible rats with fear extinction an important mediating condition. These results highlight the need for animal models of PTSD to consider stress-responsivity, as only a subset of trauma-exposed individuals develop PTSD and these individuals likely exhibit distinct neurobiological changes compared with trauma-exposed populations who are resilient to stress. This work further identifies glutamate homeostasis and mGlu5 as a target for treating relapse in comorbid PTSD-cocaine addiction.

## Introduction

Post-traumatic stress disorder (PTSD) develops in a subpopulation (15–25%) of individuals exposed to a traumatic event^1,2^. A total of 50–65% of PTSD patients suffer from substance use disorder, a rate 3–5 times higher than in the general population^1,3–5^. Comorbid PTSD and cocaine use disorder (PTSD + CUD) is especially problematic, with 43% of cocaine-dependent individuals meeting criteria for lifetime PTSD^6^ and ~34% of trauma-exposed individuals meeting criteria for lifetime CUD^7^. Cocaine addicts with comorbid PTSD are more resistant to treatment than those without a dual diagnosis^8^.

While a large body of research is devoted to studying the influence of stress on cocaine seeking, an animal model of PTSD + CUD is needed to identify unique neuroadaptations arising from, and potentially underlying, this comorbidity. Such a model should capture key features of PTSD: (1) PTSD typically arises from a single trauma, (2) not all trauma-exposed individuals develop PTSD, and (3) post-trauma anxiety symptoms (e.g. enhanced startle response) are long-lasting. When rodents are exposed to species-relevant predators or their odor (predator scent stress; PSS), a subset display long-lasting manifestations of anxiety as assessed with the elevated plus maze (EPM) and acoustic startle response (ASR)^9–13^. A strength of these models is their ethological relevance^14^ in that PSS reproduces the potentially fatal stimuli that induce PTSD in humans. Thus, we utilized the well-established PSS model of PTSD [e.g. refs.^11,15^], and found that following a single brief exposure to PSS, a subset of rats, deemed “stress-susceptible”, displayed long-term anxiety symptoms.

We first explored potential gene and hormone differences underlying different stress-induced phenotypes in order to identify targets for PTSD + CUD treatments. Since the interactions between glucocorticoid, cannabinoid and glutamate signaling are essential for the induction and maintenance of stress-induced neural plasticity^16–18^, we focused on the analysis of these signaling pathways within the stress and anxiety neural circuitry. We selected genes that either provide a read-out of stress-induced glucocorticoid activity (Glcci1^19^ and CRH^20^), or have been implicated in neurobiology of stress-related psychiatric disorders (mGlu5 receptor; CB1 receptor^21–23^). Notably, these signaling pathways have not yet been probed using a PSS model and are also implicated in cocaine seeking^24–27^. The hippocampus, medial prefrontal cortex (mPFC) and amygdala have been implicated in PSS-evoked acute, unconditioned fear responses as well as conditioned responses^28–33^.

We next assessed phenotypic differences in cocaine self-administration, instrumental extinction, and cue-primed reinstatement of cocaine seeking. We chose to use a “long-access” self-administration paradigm as it permits the assessment of escalation in cocaine intake^34^. We hypothesized that susceptible rats would exhibit a greater escalation of cocaine intake relative to stress-resilient rats and unstressed controls. We tested the antibiotic ceftriaxone for its ability to attenuate the reinstatement of cocaine seeking, as we and others have previously demonstrated its ability to do so in non-stressed rats^35–40^. Cocaine reduces, and ceftriaxone restores, the expression of two proteins (xCT and GLT-1) in the nucleus accumbens^35–40^. These proteins control glutamate homeostasis and are important regulators of cocaine relapse^37^.

While susceptible rats did not take more cocaine than controls and resilient rats, susceptible rats displayed increased lever pressing during day 1 of extinction and a cue-primed reinstatement test. Resilient rats displayed increased *mGlu5* gene expression in the amygdala and PFC. A substantial literature indicates that mGlu5 positive allosteric modulators (PAMs) enhance extinction of both drug-associated responding and fear^41–44^. This knowledge, combined with our findings of increased mGlu5 expression in resilient rats, led us to test the ability of combined treatment with the mGlu5 PAM 3-Cyano-N-(1,3-diphenyl-1 H-pyrazol-5-yl)benzamide (CDPPB) and ceftriaxone to prevent the reinstatement of cocaine seeking in susceptible rats, with fear extinction as a mediator. Fear extinction was used to maximize the translational potential of the study; fear extinction procedures are similar to exposure-based psychotherapy^45^. It also allowed us to test the hypothesis that CDPPB enhances extinction of contextual fear as well as extinction of the response previously made to obtain cocaine. Taken together, the results of these studies find neurobiological and behavioral differences in stress-susceptible rats compared to stress-resilient rats that may impact treatment of PTSD + CUD.

## Materials and methods

### Subjects

Adult male Sprague-Dawley rats (Charles River; *n* = 436) were individually housed on a reversed 12-h light cycle with lights off at 7 am. All procedures commenced 1–3 h after lights off and were approved by the Institutional Animal Care and Use Committee at the University of Florida.

### Stress induction and assessment of anxiety in initial cohort of rats

**Fig. 1:**
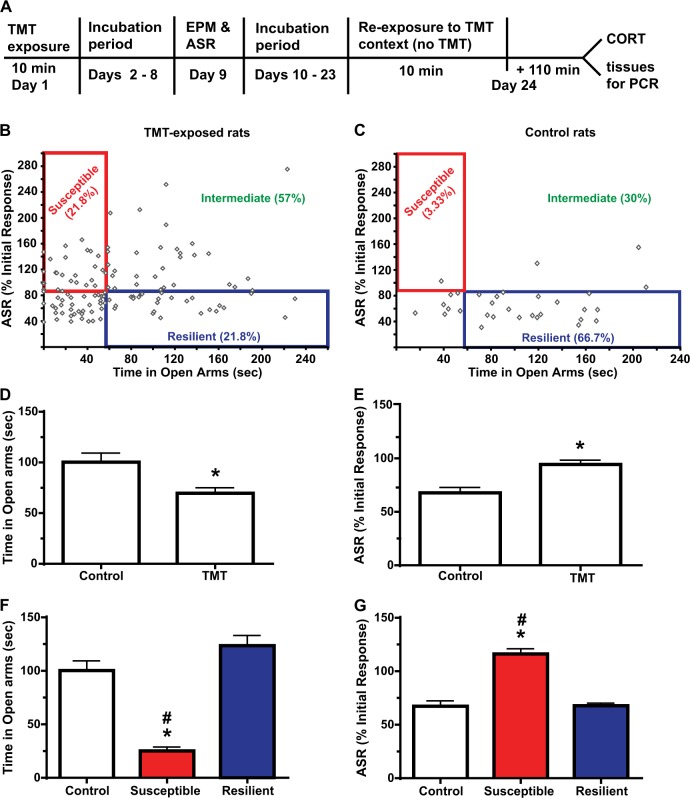
A single 10 min exposure to TMT results in three distinct anxiety phenotypes 1 week later. **a** Timeline of experimental procedures. **b** Time spent in the open arms of the EPM is plotted against startle behavior, expressed as the % habituation of initial ASR for rats exposed to TMT. A median split on both time spent in the open arms (median = 56 s) and ASR (median = 82.7% initial ASR) was used to separate TMT-exposed rats into susceptible, resilient and intermediate phenotypes. **c** Control rats did not receive TMT exposure; using the median split criteria (e.g. a cutoff of 56 s for open arm entries), only one rat was classified as stress-susceptible susceptible while 67% were classified as resilient. Comparing all TMT-exposed rats (*n* = 119) to control rats (*n* = 30) there was an effect of TMT on time spent in the open arms (**d**) and ASR habituation **e**. Upon application of the median split criteria, there were phenotypic differences in mean time spent in the open arms of the EPM (**f**) and ASR habituation **g**. For (**f**) and **g**, *n* = 30 for control, *n* = 26 each for susceptible and resilient. *p < 0.05 compared to control; #*p* < 0.05 compared to resilient. Data are presented as mean ± SEM

**Fig. 2:**
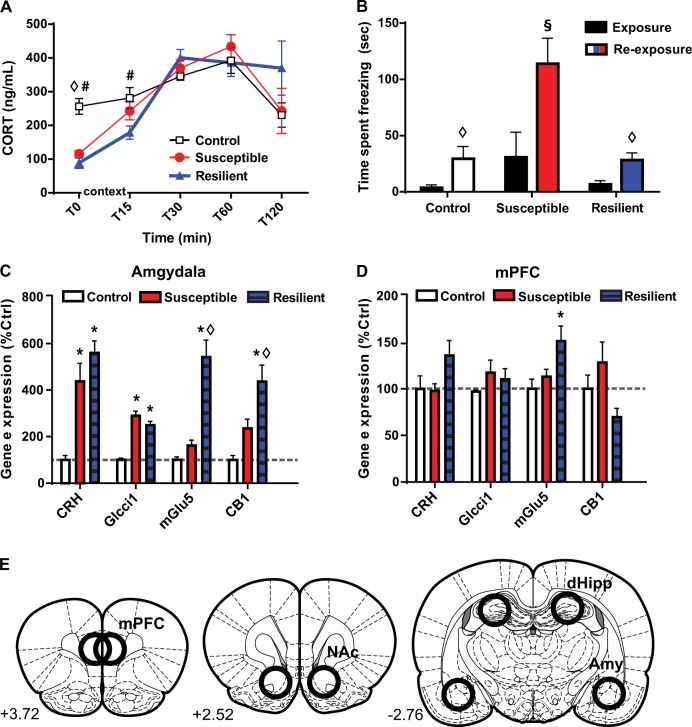
Susceptible and resilient rats differ in corticosterone, freezing, and gene expression responses upon re-exposure to the TMT context. **a** Baseline corticosterone (CORT) levels were decreased 3 weeks after TMT exposure in both susceptible and resilient rats and re-exposure to the TMT context induced a greater increase in CORT in susceptible rats **b**. Susceptible rats displayed greater freezing upon re-exposure to the TMT context relative to both freezing during the TMT exposure and to freezing in control and resilient rats during re-exposure. **c** Susceptible and resilient rats displayed similar increases in stress-related genes in the amygdala but only resilient rats displayed increases in mGlu5 and CB1 expression. **d** In the mPFC, resilient rats displayed greater *mGlu5* gene expression than controls. **e** Schematic of areas of tissue dissection. *p < 0.05 compared to control. #*p* < 0.05 compared to resilient; ◊*p* < 0.05 compared to susceptible; §*p* < 0.05 comparing exposure to re-exposure. Data are presented as mean ± SEM. *n* = 5–9/group

The first study used 150 rats that arrived in the vivarium on the same day (Figs. 1 and 2). One-twenty rats received a single 10 min exposure to the fox pheromone 2,5-dihydro-2,4,5-trimethylthiazoline (TMT) in a plexiglass chamber. Control rats (*n* = 30) were exposed to unscented bedding. Exposures occurred within 4 h of the beginning of the dark cycle, and all rats were exposed within a 12 d period. Seven days later, rats were tested in the EPM and ASR. The number of open and closed EPM arm entries and the time spent in open and closed arms were quantified using Ethovision XT 7.0 (Noldus, Leesburg, VA). Within 20 min of the EPM test, rats underwent 30 startle trials during which 110 dB white noise was presented for 40 ms followed by a variable (30–45 s) inter-trial interval. The percent initial ASR score was computed by dividing the average ASR of the last six trials by the average response of the first six trials as done previously in PSS models of PTSD [e.g. ref. ^11^]. Phenotype (susceptible, resilient, or intermediate) was assigned based on EPM and ASR criteria described in the Results section. See Supplementary Information for more details on induction and assessment. Rats then underwent a 2-week incubation period prior to re-exposure to the TMT context, assessment of freezing, plasma CORT, and gene expression (see timeline in Fig. 1a).

A baseline (T0) blood sample for CORT analysis was acquired from the tail vein within 2 h of the start of the dark cycle. This sample was followed by a 10 min re-exposure to the TMT context without TMT. Within 5 min of removal from the context, a T15 sample was collected, followed by samples at T30, T60, and T120. Samples were stored at −80 °C until analysis via radioimmunoassay. A separate cohort of rats was re-exposed to the TMT context and 2 h later, rapidly decapitated. Brains were dissected, flash frozen and stored at −80 °C for real-time quantitative reverse-transcription polymerase chain reaction (qRT-PCR) analysis. See Supplementary Information for more details on these procedures.

### Cocaine self-administration, extinction, and reinstatement

**Fig. 3:**
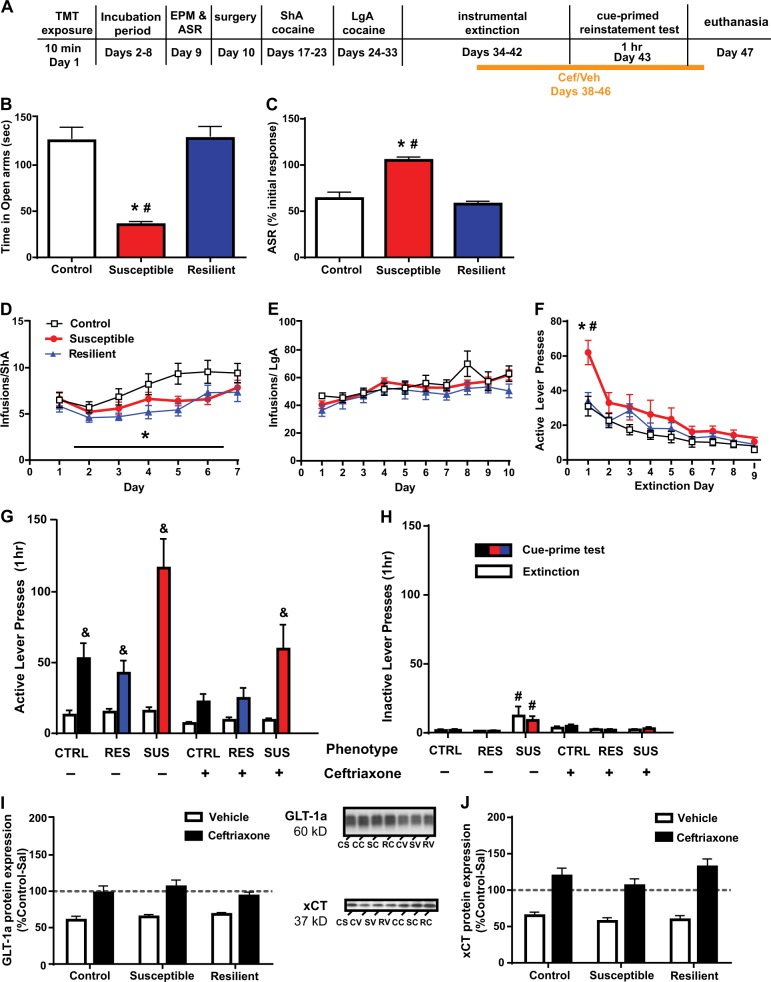
Susceptible rats demonstrate attenuated extinction learning and enhanced cue-primed reinstatement of cocaine seeking that is not prevented by ceftriaxone as it is in control and resilient rats. **a** Timeline of experimental procedures. **b**, **c** In this cohort of rats, there were phenotypic differences in mean time spent in the open arms of the EPM and ASR habituation. **d** Resilient rats self-administered less cocaine than susceptible and controls during ShA, while susceptible and controls did not differ from one another. **e** No phenotypic differences in cocaine intake were observed during LgA self-administration. **f** Presses on the previously active lever differed between phenotypes during extinction training, with the Susceptible phenotype displaying greater presses than control, resilient rats. **g** Susceptible rats displayed greater lever pressing during the reinstatement test than control, resilient phenotypes. Ceftriaxone prevented reinstatement of cocaine seeking only in control and resilient animals, while merely attenuating reinstatement in susceptible rats. **h** Inactive lever pressing did not increase during cue-primed reinstatement testing, however was greater in Veh-susceptible rats relative to resilients. **i** Nucleus accumbens GLT-1a and **j** xCT expression was reduced (relative to yoked-saline controls) to a similar degree in all cocaine self-administering rats independent of phenotype. Ceftriaxone restored GLT-1 and xCT expression in all phenotypes. * main effect of phenotype; *p < 0.05 compared to control; extinction lever pressing; #*p* < 0.05 compared to resilient; &*p* < 0.05 compared to extinction lever pressing; CS control-saline (no TMT, no cocaine); CC control-ceftriaxone; SC susceptible-ceftriaxone; RC resilient-ceftriaxone; CV control-vehicle; SV susceptible-vehicle; RV resilient-vehicle. ± = drug was/was not administered. Data are presented as mean ± SEM. *n* = 6–10/group

**Fig. 4:**
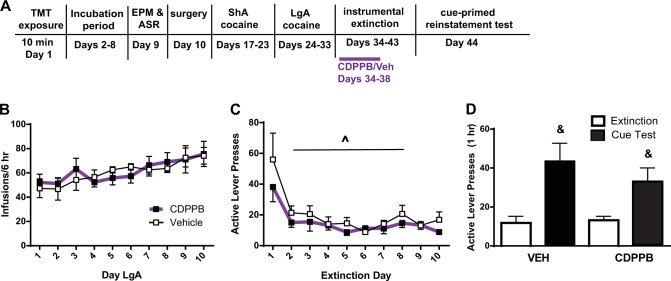
CDPPB attenuated lever pressing during extinction but did not attenuate reinstatement. **a** Timeline of experiment. **b** Infusions during LgA self-administration did not differ between groups later treated with CDPPB or Vehicle. **c** A main effect of treatment was detected, with CDPPB-treated rats exhibiting less presses on the previously active lever. **d** There was no effect of CDPPB on cue-primed reinstatement, as rats previously treated with CDPPB or vehicle reinstated lever pressing. &*p* < 0.05 compared to extinction; ^*p* < 0.05 main effect of treatment. Data are presented as mean ± SEM. *n* = 6/group

**Fig. 5:**
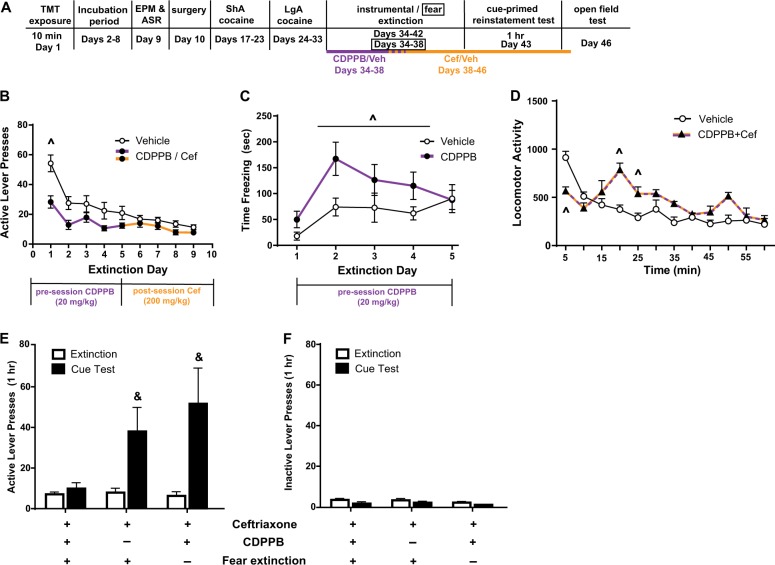
Reinstatement of cocaine seeking is prevented in susceptible rats by the combination of CDPPB treatment early in extinction and ceftriaxone later in extinction and requires fear extinction. **a** Timeline of experiment. Ceftriaxone was administered immediately following the last five extinction sessions to all rats. CDPPB was administered to a subset of rats immediately prior the first five extinction sessions; 10 min fear extinction in the TMT context followed instrumental extinction for a subset of rats. **b** CDPPB-treated rats displayed attenuated lever pressing during extinction relative to VEH-treated rats. **c** CDPPB-treated rats did not display enhanced extinction of the fear response in the TMT-context, in fact displaying greater freezing than Veh-treated rats. **d** Rats previously treated with CDPPB + ceftriaxone displayed reduced spontaneous locomotion during the first 5 minutes of an open field test, but greater locomotion later in the test. **e** The combination of CDPPB, ceftriaxone, and fear extinction according to (a) prevented the reinstatement of cocaine seeking, while the same combination without either fear extinction or CDPPB, did not prevent reinstatement. &*p* < 0.05 compared to extinction; ^*p* < 0.05 compared to CDPPB; ^*p* < 0.05 main effect of Treatment. ± = drug was/was not administered. Data are presented as mean ± SEM. *n* = 6–8/group

Rats used in the second study (cocaine self-administration and reinstatement experiment) were either controls (*n* = 25; 2 cohorts), or were exposed to TMT (*n* = 261; five cohorts arriving at different times) and categorized as susceptible or resilient (Figs. 3–5). Phenotype was assigned according to the criteria described above. One day following EPM/ASR testing, rats were implanted with chronic jugular catheters [see ref. ^37^ and Suppl. Information]. After 5–7 d recovery, rats self-administered intravenous cocaine (Research Triangle Institute; 0.33 mg/infusion/0.1 mL) using an FR-1 schedule of reinforcement. Eight control rats received “yoked-saline” infusions and were used as cocaine-naive controls for western blotting and qRT-PCR. Self-administration took place during 1 h sessions (short-access; ShA) for 7 d followed by 6 h sessions (long-access; LgA) for 10 d. An active lever press produced a cocaine infusion paired with cues (illumination of the light over the active lever and tone presentation). Following self-administration, rats underwent daily 2 h instrumental extinction sessions for 9 d during which cocaine and drug-paired cues were no longer delivered upon lever pressing. During extinction, rats were treated with ceftriaxone (Cef; 200 mg/kg IP) or vehicle (Veh; 0.9% saline) and/or CDPPB (30 mg/kg SC) or vehicle (0.5% Tween in saline) according to the timelines in Figs. 3a, 4a, and 5a. This dose of ceftriaxone was chosen and administered after extinction sessions as this regimen has previously been demonstrated to attenuate the reinstatement of cocaine seeking 24 h after the last ceftriaxone injection^36,37,39^. CDPPB was administered 20 min prior to instrumental extinction, in accordance with its 4.4 h half-life^46^. This dose of CDPPB enhances extinction of conditioned drug responses^42,43^ and a dose of 20 mg/kg enhances contextual fear extinction^44^. A higher dose of CDPPB (60 mg/kg) does not inhibit locomotion in rats^41^. A subset of CDPPB and vehicle-treated rats experienced contextual fear extinction for 10 min/day, immediately following instrumental extinction sessions 1–5. This timing was chosen to avoid potential effects of context-induced anxiety on instrumental extinction. Rats underwent a 1 h reinstatement test wherein active lever presses yielded drug-paired cues. Rats in the CDPPB + ceftriaxone and vehicle groups received a single open-field test of locomotion. Ceftriaxone and vehicle injections continued and 4 d later, rats were decapitated. Brains were extracted and stored at −80 °C for qRT-PCR and western blotting.

### Gene expression and western blotting analysis

Amygdala, mPFC, dorsal hippocampus (dHipp), and nucleus accumbens (NAc) tissue was micropunched in a cryostat according to the rat brain atlas^47^; Fig. 2e. Tissue was processed for qRT-PCR measurement (see Supplementary Information and Table 1). For the self-administration study, one hemisphere was used for qRT-PCR and the second for western blotting. GLT-1a and xCT immunoreactivity was analyzed in the total tissue lysate with calnexin as a loading control. See Suppl. Information for details.

**Table 1:** PCR primers

Primer	Forward	Reverse
**CB1**	TTCAGGTAGCGGGGCATTTT	GCCACAGCTCCGATTCTACA
**mGlu5**	CAGCAGACCTCGGTGTTCCA	CCTTGGCATTTTTCACCTCGG
**CRH**	CCGCAGCCGTTGAATTTCTT	CTTCACCCATGCGGATCAGA
**Glcci1**	GGGTCCAGGTCAGTCCCTAT	CGCTCTTGGACAGAAGGTGT

### Statistical analysis

GraphPad Prism (version 6.0) and SPSS (version 12.0) were used for statistical analyses, with the alpha level set at *p* ≤ 0.05. ASR and EPM data were first compared between TMT and control groups using *t*-tests. One-way analyses of variance (ANOVAs) were used to assess phenotypic differences in EPM and ASR behavior and gene expression; Brown-Forsythe tests confirmed equal variance. CORT concentrations, freezing, self-administration, extinction, and reinstatement data were analyzed with mixed-factorial 2- or 3-way ANOVAs, with phenotype or treatment group (e.g. ceftriaxone, vehicle) as between-subjects factors and time as a within-subjects factor. Significant interactions were followed by Tukey’s (SPSS) or Sidak’s (Prism) post hoc analyses, with corrections for multiple comparisons. Bivariate (Pearson’s) correlations were used to assess the association between the ASR/EPM measures and cocaine seeking. Fisher’s exact test was used to compare the frequency of phenotypes in different rat cohorts. This test is suitable for data sets with unequal sample sizes^48^. For immunoblotting, the integrated density of individual protein bands was divided by the density of calnexin immunoreactivity within the same sample. Treatment groups were normalized to the yoked-saline group and compared using two-way multifactorial ANOVAs, with phenotype and treatment (ceftriaxone or vehicle) as between-subjects factors.

## Results

### Characterization of the effects of TMT on anxiety-like behavior

One-twenty rats were exposed to TMT while 30 rats experienced the control condition. One TMT-exposed rat escaped the EPM and was excluded from analysis. The number of open arm entries was reduced by TMT [*t*_(1,147)_ = 3.779, *p* = 0.0002, not shown]. Similarly, the TMT-exposed group spent less time in the open arms [*t*_(1,147)_ = 2.710, *p* = 0.0075; Fig. 1d] and exhibited reduced habituation of the ASR [*t*_(1,147)_ = 3.285, *p* < 0.0013; Fig. 1e]. A median split on time spent in the open arms and ASR habituation scores yielded a median of 56 s for the EPM and 82.7% for the ASR. Rats were classified as stress-susceptible (susceptible) if time spent in the open arms was below the median and ASR habituation was above the median. Rats were classified as stress-resilient (resilient) if they fell above the median for time spent in the open arms and below the median for ASR habituation. This resulted in 21.8% TMT-exposed rats classified as susceptible, and an equal number classified as resilient (Fig. 1b). The remaining 56% rats met criteria for resilience/susceptible only in one test, and were classified as Intermediate. Using the same criteria, only 1 of 30 controls was classified as susceptible and 66.7% were resilient (Fig. 1c). Time spent in the open arms differed by Phenotype [*F*_(2,79)_ = 40.13, *p* < 0.0001; Fig. 1f]; the susceptible group spent less time in the open arms than control and resilient rats. ASR habituation also differed by Phenotype, [*F*_(2,79)_ = 40.14, *p* < 0.0001; Fig. 1g], with the susceptible phenotype displaying less ASR habituation relative to both control and resilient rats. For all rats exposed to TMT (*n* = 119), there was a trend for percent ASR habituation to correlate with time spent in the open arms (*r* = −0.1692, *p* = 0.06), suggesting that these two behavioral tests assess anxiety in a consistent manner. The prevalence of the three phenotypes differed between control and TMT-exposed rats (*p* < 0.0001, Fisher’s test).

### Long-term effects of phenotype on corticosterone and contextual fear

Three weeks following TMT exposure or the control condition, rats were re-exposed to the TMT context without TMT and blood collected from the tail vein for CORT analysis. While a phenotype × time interaction [*F*_(8,60)_ = 3.594, *p* = 0.0018] was detected when examining all 5 sampling times, by T30 CORT levels were raised in all groups, a likely consequence of repeated sampling from the tail vein (Fig. 2a). Control CORT concentrations increased from T0 to T30 and T60 (*p* < 0.001), but did not change from T0 to T15. Thus, at T15, tail vein sampling was not inducing CORT release. Comparison of only T0 and T15 samples revealed a significant phenotype × time interaction [*F*_(2,15)_ = 5.913, *p* = 0.0128]. T0 CORT concentration in both TMT-exposed groups was reduced relative to controls (*p* < 0.05). From T0 to T15, CORT concentration increased in both susceptible and resilient rats (*p* < 0.05), but the CORT concentration of resilient rats remained lower than controls.

Comparing time spent freezing during the TMT exposure and context re-exposure revealed a significant phenotype × time interaction [*F*_(2,14)_ = 6.076, *p* = 0.0126; Fig. 2b]. During the re-exposure, susceptible rats displayed greater freezing than control and resilient rats. Only susceptible rats increased freezing from exposure to re-exposure (*p* < 0.001).

### Effects of phenotype on gene expression

In the amygdala, CRH gene product differed by phenotype [*F*_(2,20)_ = 15.70, *p* < 0.0001; Fig. 2c]; both susceptible and resilient rats displayed greater product than controls. A similar pattern was observed for Glcci1 [*F*_(2,19) =_ 45.54, *p* < 0.0001]. However, mGlu5 gene product differed by Phenotype [*F*_(2,19)_ = 36.09, *p* < 0.0001], with resilient rats displaying greater expression than both susceptible rats and controls. A similar pattern was found for CB1 expression [*F*_(2,15)_ = 12.51, *p* = 0.0006].

*CRH* and *Glcci1* gene product expression did not differ by phenotype in the mPFC (Fig. 2d). As in the amygdala, mGlu5 gene expression was altered by Phenotype [*F*_(2,18)_ = 5.007, *p* = 0.0187], with resilient rats displaying greater expression than controls. In the dHipp, only Glcci1 gene product was changed [*F*_(2,20)_ = 5.589, *p* = 0.0118], with both susceptible and resilient phenotypes displaying greater Glcci1 compared to control (*p* < 0.05; not shown). No effects were observed in the NAc (not shown).

### Effects of post-trauma phenotype on cocaine seeking and extinction learning

Phenotypic differences in time spent in the open arms [*F*_(2,44)_ = 24.11, *p* < 0.001; Fig. 3b] and ASR habituation [*F*_(2,44)_ = 27.05, *p* < 0.0001; Fig. 3c] were observed in the rats used for the self-administration experiment. Within the three cohorts of rats used to generate Fig. 3 data, the incidence of the susceptible phenotype was: 21%, 14%, 18% and resilient was: 31%, 22%, 21%. There was no difference in prevalence of phenotypes between TMT-exposed rats in different cohorts. Within the 25 control rats, the proportion was susceptible: 4%, resilient: 60%. The frequency of phenotypes did not differ between the two cohorts of control rats, but did between control and TMT-exposed rats used for Fig. 3 (*p* < 0.0001, Fisher’s test).

A one-way ANOVA found a main effect of Phenotype on infusions during ShA; resilient rats self-administered less cocaine than control and susceptible rats, while susceptible and controls did not differ from one another [*F*_(2,44)_ = 8.182, *p* = 0.0010; Fig. 3d]. During LgA, all groups escalated cocaine intake, evidenced by a significant main effect of Time [*F*_(9,369)_ = 12.60, *p* < 0.001; Fig. 3e), with no phenotypic differences in intake. There was no effect of phenotype (or phenotype × time interaction) on active or inactive lever pressing during self-administration, or inactive lever presses during extinction (Fig. S1a-e). A phenotype × time interaction was detected for presses on the previously active lever during extinction [*F*_(16,352)_ = 2.629, *p* = 0.0007, Fig. 3f]. On day 1 of extinction, susceptible rats displayed greater lever presses than all control and resilient rats (*p* < 0.001).

Three-way ANOVAs were conducted to ensure that self-administration behavior did not differ between groups later treated with ceftriaxone/vehicle. Only infusions during ShA differed, due to the abovementioned decrease in infusions in resilient rats [treatment × phenotype: *F*(1,2) = 4.894, *p* = 0.012]. However, total number of infusions attained during self-administration did not differ (*p* > 0.05). There was also no effect of ceftriaxone on inactive or active lever pressing during extinction (Fig. S3G). Analysis of active lever presses during the cue test (Fig. 3g) revealed main effects of treatment [*F*_(1,41)_ = 10.056, *p* = 0.003] and phenotype [*F*_(2,41)_ = 11.728, *p* = 0.000]. A treatment × time interaction was found [*F*_(2,41)_ = 10.056, *p* = 0.003], as ceftriaxone only attenuated lever pressing during test and not extinction. Post hoc analyses revealed that as a whole, susceptible rats displayed greater lever presses during the test than control and resilient rats (*p* = 0.000). Neither a phenotype × treatment × time nor a phenotype × treatment interaction was detected, indicating that ceftriaxone attenuated reinstatement similarly in all phenotypes. Reinstatement of the cocaine seeking response is defined as an increase in presses on the previously active lever from extinction to test. Thus, we followed a significant main effect of Time [*F*_(1,41)_ = 87.45, *p* = 0.000] and a phenotype × time interaction [*F*_(2,41)_ = 14.249, *p* = 0.000] with post hoc comparisons of extinction and test lever pressing within each group (not between groups). All Veh-treated groups reinstated lever pressing (*p* < 0.05). While ceftriaxone was able to prevent cue-primed reinstatement in control and resilient rats (e.g. extinction vs. reinstatement *p* > 0.05), it was not able to do so in susceptible rats (extinction vs. test, *p* < 0.001). Inactive lever pressing did not increase during the test, as no main effect of Time, nor three-way interaction, was detected (Fig. 3h). However, a phenotype × treatment interaction [*F*_(2,41)_ = 4.291, *p* = 0.020] was detected. Post hoc tests found that susceptible-Veh rats pressed more on the inactive lever during the last two days of extinction and reinstatement compared to resilient-Veh and resilient-ceftriaxone groups only (*p* < 0.05).

### Correlations between anxiety and instrumental behavior

A number of significant correlations between anxiety and instrumental behaviors were detected when examining all 47 rats depicted in Fig. 3f, g. ASR habituation correlated with lever pressing on day 1 extinction (*r* = 0.3834, *p* = 0.0078; Fig. S2A) and reinstatement (*r* = 0.5799, *p* < 0.0001; Fig. S2B). ASR habituation also correlated with total infusions attained during ShA (*r* = 0.3500, *p* = 0.0159), with less reactivity predictive of reduced cocaine intake (Fig. S4C). Time spent in the open arms negatively correlated with Extinction day 1 pressing (*r* = −0.4333, *p* = 0.0023; Fig. S2D), indicating that anxiety-like behavior in the EPM predicts lever pressing on the first day of extinction. Time spent in the open arms correlated with lever pressing during reinstatement only in vehicle-treated rats (*r* = −0.4225, *p* = 0.0446; not shown), but not when ceftriaxone-treated rats were included. Thus, unlike with ASR habituation, time spent in the open arms does not predict the ability of ceftriaxone to attenuate reinstatement.

The same correlational analyses were conducted only in controls. ASR behavior did not correlate with either extinction or reinstatement lever pressing. Time spent in the open arms was not correlated with extinction behavior, but was positively correlated with active lever presses during reinstatement (*r* = 0.564, *p* = 0.035; not shown).

### GLT-1, xCT, and mGlu5 expression after cocaine self-administration, extinction, reinstatement

Protein and gene expression were both compared to control rats that received yoked-saline injections. GLT-1 protein expression differed by treatment [*F*_(1,37)_ = 51.27 *p* < 0.0001; Fig. 3i], but not phenotype. No treatment × phenotype interaction was observed. Similar results were found for xCT [*F*_(1,37)_ = 73.37; *p* < 0.001; Fig. 3j]. Thus, cocaine reduced expression of both GLT-1 and xCT and ceftriaxone restored expression of these proteins, independent of phenotype. mGlu5 gene expression differed by Group in the amygdala [*F*_(3,23)_ = 4.584, *p* = 0.0117; Fig. S1F], mPFC [*F*_(3,25)_ = 3.350, *p* = 0.0350], and NAc [*F*_(3,26)_ = 3.830, *p* = 0.0214]. Relative to controls receiving yoked-saline, resilient rats displayed greater mGlu5 in the mPFC and susceptible rats displayed less in the NAc. In the amygdala, controls that self-administered cocaine displayed less mGlu5 than both resilients and yoked-saline controls.

### Manipulating mGlu5 activity and extinction and reinstatement of cocaine seeking

We next tested the ability of CDPPB to enhance instrumental extinction and subsequently attenuate cue-primed reinstatement of drug-seeking in Intermediate rats. We hypothesized that we would be more likely to detect an effect on reinstatement in this population, and if so, we would later test CDPPB in susceptible rats. CDPPB (or Veh) was administered 20 min prior to the first 5 instrumental extinction sessions (Fig. 4a), as in our experience the majority of extinction learning occurs during this time. There were no differences in cocaine infusions (Fig. 4b), active (Fig. S3B, D) or inactive (Fig. S3C, E) lever presses during self-administration. During extinction training, CDPPB reduced pressing on the previously active lever relative to vehicle-treated rats [*F*_(1,90)_ = 8.618, *p* = 0.0042; Fig. 4c). However, CDPPB administered during the first 5 days of extinction did not attenuate later reinstatement of cocaine seeking; both CDPPB and VEH rats reinstated, evidenced by a significant main effect of Time [*F*_(1,10)_ = 16.09, *p* = 0.0025; Fig. 4d], but not treatment. No treatment × time interaction was observed. There was no effect of time or treatment on inactive lever presses during the test (Fig. S3F).

Based on the results in Fig. 4, we hypothesized that CDPPB administered to susceptible rats would enhance extinction but would not reduce reinstatement of cocaine seeking. Thus, we administered CDPPB during the first 5 days of extinction training and ceftriaxone immediately following the last 5 extinction trials, (as in refs. ^37–39^ and Fig. 3). A subset of CDPPB + ceftriaxone rats underwent contextual fear extinction during the first 5 days of instrumental extinction training (see timeline in Fig. 5a). Finally, we also included rats receiving ceftriaxone and contextual fear extinction, but no CDPPB. As CDPPB alone did not attenuate reinstatement in Intermediate rats (Fig. 4d), we did not test its effectiveness in susceptible rats. There were no differences in self-administration behavior between rats later assigned to treatment groups (Fig. S4B–G). The frequency of the susceptible phenotype in the two cohorts of rats used for Fig. 5 data was 17 and 20%; resilient: 21 and 29%. There was no difference in frequency of any phenotype in the 6 TMT-exposed cohorts used throughout the studies presented here.

CDPPB reduced pressing on the previously active lever (but not inactive, Fig. S4H) during instrumental extinction (Fig. 5b; *F*_(8,256)_ = 3.214, *p* = 0.0017). In contrast to our hypothesis, CDPPB-treated rats displayed more freezing than vehicle-treated rats [*F*_(1,13)_ = 4.868, *p* = 0.0460, Fig. 5c]. As freezing did not differ between groups on day 1, CDPPB did not increase fear, but rather the consolidation of fear-related memories. A group × time interaction was detected for presses on the previously active lever during extinction and reinstatement [Fig. 5d, *F*_(2,18)_ = 3.803, *p* = 0.0419]. Rats treated with CDPPB + ceftriaxone without fear extinction reinstated cocaine seeking (post hoc test comparing extinction to test, *p* = 0.01), as did rats receiving fear extinction and ceftriaxone but not CDPPB (*p* = 0.05). CDPPB administered prior to instrumental and fear extinction for 5 days, followed by 5 days of ceftriaxone prevented cue-primed reinstatement (*p* > 0.05). This effect was not due to locomotor sedation as this group displayed greater locomotion than vehicle-treated rats in a test of spontaneous locomotion [*F*_(11,120)_ = 6.141, *p* < 0.0001; Fig. 5d], in agreement with our previous work in rats receiving 5 days of ceftriaxone^36^. CDPPB + ceftriaxone rats displayed less locomotion than the vehicle group during the first 5 minutes but increased locomotion later in the test. There was a main effect of time on inactive lever pressing during the reinstatement test [*F*_(1,17)_ = 11.21, *p* = 0.0038; Fig. 5f]. Post hoc tests found pressing decreased from extinction to test, in contrast with active lever pressing.

## Discussion

Here we utilized the PSS model of PTSD and a median split analysis of EPM and ASR data to separate rats into stress-susceptible and stress-resilient phenotypes. There are caveats to using median splits, such as inclusion of rats falling immediately below or above the median. However, we propose that the requirement to meet criteria on two tests increases the probability that true anxiety phenotypes are detected. The incidence of each phenotype remained stable throughout the 6 cohorts of TMT-exposed rats utilized. The use of this phenotyping method is also validated by the continued presence of phenotypic differences weeks later across multiple measures, including gene expression, context-induced freezing, extinction, and reinstatement of cocaine seeking. Only susceptible rats showed conditioned fear responses 2-weeks after TMT exposure. There are reports that TMT does not produce such fear responses (e.g. refs. ^49–51^), however these reports tested subjects 24 h after TMT and examined the entire population of TMT-exposed rats. Here, resilient rats did not display conditioned fear responses.

We are the first to report that resilience to stress is accompanied by enhanced expression of the plasticity-related genes mGlu5 and CB1 in the amygdala and mGlu5 in the mPFC weeks after a stressor. Both proteins are important for fear-related plasticity in the amygdala^52^ and PFC^53^, indicating that an upregulation of the machinery that underlies plasticity could promote resilience to stress. Conversely, constitutive genetic deletion of CB1 or mGlu5 attenuates fear extinction^52,54^. Increased cortical mGlu5 and CB1 forebrain binding has been recently reported in subjects with a PTSD diagnosis, suggesting clinical relevance of manipulations targeting mGlu5 and/or CB1 receptors^23,55^. However, mGlu5 binding in trauma-exposed, resilient patients was not assessed. Thus, analysis of receptor binding capacity in rodent models of PSS should be conducted in future studies to allow for comparison and cross-interpretation of rodent and human data.

Susceptible rats displayed greater pressing on the cocaine lever during extinction and cue-primed reinstatement than did control and resilient rats. Escalation of cocaine intake in LgA self-administration models is followed by increased cocaine-primed reinstatement^34,56^. Here we found increased cue-primed reinstatement in susceptible rats that did not have increased intake, consistent with other reports finding dissociation between escalation and persistent cue-elicited cocaine seeking^57,58^. Behavior in the EPM and ASR correlated with extinction and reinstatement responding. Interestingly, in control rats not exposed to TMT, reduced anxiety in the EPM was associated with greater active lever presses during reinstatement, the opposite relationship observed in TMT-exposed rats. This data indicate that in the absence of PSS exposure, baseline anxiety does not predict cocaine seeking.

Ceftriaxone similarly attenuated cued-seeking in all phenotypes. In agreement with previous work^37^, ceftriaxone prevented the reinstatement of cocaine seeking in control and resilient rats, but was unable to do so in susceptible rats. It is possible that a higher dose of ceftriaxone could prevent reinstatement in susceptible rats. However, to our knowledge, the dose used here is the highest dose used in rats across a variety of models of neurological diseases. During the last 2 days of extinction and reinstatement testing, inactive lever pressing was higher in vehicle-treated susceptible rats. As this pattern was not present during self-administration or early extinction, and pressing was still a fraction of that on the active lever, the relevance of this finding is unknown. It is possible that increased drug-seeking was manifested as pressing both available levers in the chamber.

Confirming the importance of mGlu5 in mediating resilience to stress and addiction in animal models, the mGlu5 PAM CDPPB administered early in instrumental extinction training followed by ceftriaxone in the last 5 days of extinction prevented cue-primed reinstatement. This effect was dependent on fear extinction, as administration of CDPPB + ceftriaxone in the absence of fear extinction did not prevent reinstatement, nor did ceftriaxone combined with fear extinction in the absence of CDPPB. While fear responses did not extinguish in CDPPB-treated rats, such exposure was therapeutic in the case of cued cocaine seeking. We propose that mGlu5 PAM interacts with contextual and instrumental extinction to remodel cocaine-seeking circuits to make them amenable to ceftriaxone treatment. Such treatment was not necessary in resilient rats, as this phenotype displayed elevated mGlu5 expression relative to susceptible rats. Admittedly, more parametric work is necessary in order to understand the timing and dosing conditions of combined CDPPB, ceftriaxone and fear extinction. This will be a goal of future studies. Because the model of cue-primed reinstatement of cocaine seeking requires instrumental extinction, we did not test CDPPB + ceftriaxone in the absence of instrumental extinction. Future studies should do so, especially in light of the present observations regarding the necessity of fear extinction.

The inability of ceftriaxone alone to prevent reinstatement in susceptible rats did not stem from greater cocaine-induced reductions in NAc xCT or GLT-1, or from a reduced ability of ceftriaxone to increase GLT-1 and xCT. This is consistent with our finding that GLT-1 over-expression in the NAc is necessary, but not sufficient, to attenuate cue-primed reinstatement of cocaine seeking^59^. Phenotypic differences in mGlu5 were detected in the amygdala, mPFC and NAc following cocaine self-administration, potentially indicating that reduced mGlu5 in susceptible rats accounts for persistent drug-seeking. While these results were consistent with those in the cocaine-naive rats, they do not explain differences in cocaine-seeking behavior, as control and resilient rats do not differ in reinstatement. However, these tissues were collected after the reinstatement test, and may not reflect expression at the time the rats were placed into the instrumental chamber for the test. Increased cue-primed reinstatement may be due to lack of plasticity in the amygdala, as this brain region is necessary for cue-primed reinstatement of cocaine seeking^60,61^. Exaggerated amygdala activation among individuals with PTSD occurs in response to trauma-related stimuli and negative stimuli unrelated to trauma^62,63^.

In agreement with previous reports^41,43^, CDPPB reduced instrumental responding during extinction. We are the first to show that it does not affect cued cocaine seeking 5 days after cessation of administration. In contrast to results utilizing a conditioned footshock model^44^, CDPPB did not enhance contextual fear extinction in susceptible rats. This discrepancy may be due to unique post-cocaine adaptations in mGlu5 expression or signaling. Alternatively, CDPPB not only enhances the extinction of, but also the acquisition and consolidation of, fear memories; CDPPB administered prior to contextual fear conditioning increases subsequent freezing^44^. CDPPB did not alter freezing on the first day of administration, and thus it likely enhanced consolidation of contextual fear-related memories prompted by re-exposure to the trauma context. Freezing among vehicle-treated susceptible rats did not decline over 5 days, consistent with reports that PTSD patients do not extinguish memories related to their own trauma^64^, nor do they extinguish fear memories unrelated to the trauma^65^. Our model captures both of these extinction deficits.

Susceptible and resilient rats showed similar reductions in basal CORT concentrations, increased *CRH* and *Glcci1* gene expression in the amygdala, and increased Glcci1 in the dHipp. Thus, behavioral resilience in our model does not stem from differences in basal CORT or CORT/stress-responsive gene products^19,66^ in these brain regions 3 weeks after PSS. Increased dHipp CRH mRNA is found in Susceptible, but not resilient, rats 8 days after PSS^67^, at a time when susceptible rats have higher basal CORT concentrations relative to resilient rats^68,69^. Here, CORT concentrations were assessed during the “early waking” phase, when CORT levels are at their highest, ~250 ng/mL in unstressed Sprague-Dawley rats^70^. TMT-exposed rats displayed reduced CORT, in agreement with work in humans finding that morning CORT levels in PTSD patients are lower^71^ than controls (see also ref. ^72^). Resilient rats displayed an attenuated CORT response following TMT context re-exposure. As activity at the CB1 receptor negatively modulates the HPA axis^73^, this effect may be due to the observed increase in amygdala CB1 expression. In agreement with our results, PTSD-individuals show increased CORT responses to stressors compared to trauma-exposed and trauma-unexposed individuals without PTSD^74,75^. Thus, the observed basal and stress-evoked changes in CORT here are consistent with those observed in human PTSD patients, and thus support our method of classifying rats as susceptible and resilient. A recent report using the PSS model and separating rats into those susceptible and resilient to long-term effects of TMT found increased basal CORT in susceptible rats^76^. Sampling was done in the late waking phase, indicating the potential for changes in diurnal fluctuations in CORT levels in susceptible rats.

An extensive literature explores interactions between stress and cocaine addiction and several recent papers find long-term increases in drug-seeking after an acute stressor^77–79^. However, this is the first study to separate stress-resilient from susceptible rats to find that cocaine seeking is altered by the presence (and absence) of TMT-induced anxiety. Screening rats for stress-responsivity is crucial to the study of comorbid PTSD + CUD, as only a subset of trauma-exposed individuals develop PTSD and thus likely exhibit distinct neurobiological changes from those who are resilient. Our results from this model are largely in agreement with the human literature, highlighting its translational potential. This work further identifies mGlu5 and glutamate homeostasis as targets for treating both cocaine relapse and PTSD symptoms in comorbid populations, however caution should be used in combining mGlu5 PAMs with exposure therapy. Finally, we propose that in order to develop treatments to reduce cocaine seeking in a comorbid population, models must consider stress-resiliency and susceptibility.

## Electronic supplementary material

Suppl Figure 1

Suppl Figure 2

Suppl Figure 3

Suppl Figure 4

## References

[1] Breslau N, Davis GC, Schultz LR (2003). Posttraumatic stress disorder and the incidence of nicotine, alcohol, and other drug disorders in persons who have experienced trauma. Arch. Gen. Psychiatry.

[2] Breslau N, Davis GC, Andreski P, Peterson E (1991). Traumatic events and posttraumatic stress disorder in an urban population of young adults. Arch. Gen. Psychiatry.

[3] Perkonigg A, Kessler RC, Storz S, Wittchen HU (2000). Traumatic events and post-traumatic stress disorder in the community: prevalence, risk factors and comorbidity. Acta Psychiatr. Scand..

[4] Pietrzak RH, Goldstein RB, Southwick SM, Grant BF (2011). Prevalence and Axis I comorbidity of full and partial posttraumatic stress disorder in the United States: results from wave 2 of the national epidemiologic survey on alcohol and related conditions. J. Anxiety Disord..

[5] Mills KL, Teesson M, Ross J, Peters L (2006). Trauma, PTSD, and substance use disorders: findings from the Australian National Survey of Mental Health and well-being. Am. J. Psychiatry.

[6] Back S (2000). Cocaine dependence with and without post-traumatic stress disorder: a comparison of substance use, trauma history and psychiatric comorbidity. Am. J. Addict..

[7] Khoury L, Tang YL, Bradley B, Cubells JF, Ressler KJ (2010). Substance use, childhood traumatic experience, and Posttraumatic Stress Disorder in an urban civilian population. Depress Anxiety.

[8] Coffey SF (2002). Trauma and substance cue reactivity in individuals with comorbid posttraumatic stress disorder and cocaine or alcohol dependence. Drug Alcohol Depend..

[9] Adamec RE, Shallow T (1993). Lasting effects on rodent anxiety of a single exposure to a cat. Physiol. Behav..

[10] Mesches MH, Fleshner M, Heman KL, Rose GM, Diamond DM (1999). Exposing rats to a predator blocks primed burst potentiation in the hippocampus in vitro. J. Neurosci..

[11] Cohen H (2004). Setting apart the affected: the use of behavioral criteria in animal models of post traumatic stress disorder. Neuropsychopharmacology.

[12] Adamec R, Walling S, Burton P (2004). Long-lasting, selective, anxiogenic effects of feline predator stress in mice. Physiol. Behav..

[13] Adamec R, Head D, Blundell J, Burton P, Berton O (2006). Lasting anxiogenic effects of feline predator stress in mice: sex differences in vulnerability to stress and predicting severity of anxiogenic response from the stress experience. Physiol. Behav..

[14] Clinchy M (2010). The neurological ecology of fear: insights neuroscientists and ecologists have to offer one another. Front. Behav. Neurosci..

[15] Cohen H, Kozlovsky N, Alona C, Matar MA, Joseph Z (2012). Animal model for PTSD: from clinical concept to translational research. Neuropharmacology.

[16] Popoli M, Yan Z, McEwen BS, Sanacora G (2011). The stressed synapse: the impact of stress and glucocorticoids on glutamate transmission. Nat. Rev. Neurosci..

[17] Hill MN, McEwen BS (2010). Involvement of the endocannabinoid system in the neurobehavioural effects of stress and glucocorticoids. Prog. Neuropsychopharmacol. Biol. Psychiatry.

[18] Akirav I (2013). Cannabinoids and glucocorticoids modulate emotional memory after stress. Neurosci. Biobehav. Rev..

[19] Tantisira KG (2011). Genomewide association between GLCCI1 and response to glucocorticoid therapy in asthma. N. Engl. J. Med..

[20] Thompson BL, Erickson K, Schulkin J, Rosen JB (2004). Corticosterone facilitates retention of contextually conditioned fear and increases CRH mRNA expression in the amygdala. Behav. Brain. Res..

[21] Esterlis I, Holmes SE, Sharma P, Krystal JHampDeLorenzoC (2018). Metabotropic glutamatergic receptor 5 and stress disorders: knowledge gained from receptor imaging studies.. Biol. Psychiatry.

[22] Hill MN, Campolongo P, Yehuda R, Patel S (2018). Integrating endocannabinoid signaling and cannabinoids into the biology and treatment of posttraumatic stress disorder. Neuropsychopharmacology.

[23] Holmes SE (2017). Altered metabotropic glutamate receptor 5 markers in PTSD: In vivo and postmortem evidence. Proc. Natl Acad. Sci. USA.

[24] Zhou Y, Spangler R, Ho A, Kreek MJ (2003). Increased CRH mRNA levels in the rat amygdala during short-term withdrawal from chronic “binge” cocaine. Brain. Res. Mol. Brain Res..

[25] Mantsch JR (2007). Daily cocaine self-administration under long-access conditions augments restraint-induced increases in plasma corticosterone and impairs glucocorticoid receptor-mediated negative feedback in rats. Brain Res..

[26] McReynolds JR (2016). CB1 receptor antagonism blocks stress-potentiated reinstatement of cocaine seeking in rats. Psychopharmacology.

[27] Li X (2018). mGluR5 antagonism inhibits cocaine reinforcement and relapse by elevation of extracellular glutamate in the nucleus accumbens via a CB1 receptor mechanism. Sci. Rep..

[28] Wallace KJ, Rosen JB (2001). Neurotoxic lesions of the lateral nucleus of the amygdala decrease conditioned fear but not unconditioned fear of a predator odor: comparison with electrolytic lesions. J. Neurosci..

[29] Müller M, Fendt M (2006). Temporary inactivation of the medial and basolateral amygdala differentially affects TMT-induced fear behavior in rats. Behav. Brain Res..

[30] Fitzpatrick CJ, Knox D, Liberzon I (2011). Inactivation of the prelimbic cortex enhances freezing induced by trimethylthiazoline, a component of fox feces. Behav. Brain Res..

[31] Takahashi LK, Chan MM, Pilar ML (2008). Predator odor fear conditioning: current perspectives and new directions. Neurosci. Biobehav. Rev..

[32] Cohen H, Kozlovsky N, Matar MA, Zohar J, Kaplan Z (2014). Distinctive hippocampal and amygdalar cytoarchitectural changes underlie specific patterns of behavioral disruption following stress exposure in an animal model of PTSD. Eur. Neuropsychopharmacol..

[33] Cohen H, Vainer E, Zeev K, Zohar J, Mathé AA (2018). Neuropeptide S in the basolateral amygdala mediates an adaptive behavioral stress response in a rat model of posttraumatic stress disorder by increasing the expression of BDNF and the neuropeptide YY1 receptor. Eur. Neuropsychopharmacol..

[34] Knackstedt LA, Kalivas PW (2007). Extended access to cocaine self-administration enhances drug-primed reinstatement but not behavioral sensitization. J. Pharmacol. Exp. Ther..

[35] Sari Y, Smith KD, Ali PK, Rebec GV (2009). Upregulation of GLT1 attenuates cue-induced reinstatement of cocaine-seeking behavior in rats. J. Neurosci..

[36] Knackstedt LA, Melendez RI, Kalivas PW (2010). Ceftriaxone restores glutamate homeostasis and prevents relapse to cocaine seeking. Biol. Psychiatry.

[37] LaCrosse AL (2017). Contrasting the role of xCT and GLT-1 upregulation in the ability of ceftriaxone to attenuate the cue-induced reinstatement of cocaine seeking and normalize AMPA receptor subunit expression. J. Neurosci..

[38] Sondheimer I, Knackstedt LA (2011). Ceftriaxone prevents the induction of cocaine sensitization and produces enduring attenuation of cue- and cocaine-primed reinstatement of cocaine-seeking. Behav. Brain Res..

[39] Bechard AR, Hamor PU, Schwendt M, Knackstedt LA (2018). The effects of ceftriaxone on cue-primed reinstatement of cocaine-seeking in male and female rats: estrous cycle effects on behavior and protein expression in the nucleus accumbens. Psychopharmacology.

[40] Fischer KD, Houston ACW, Rebec GV (2013). Role of the major glutamate transporter GLT1 in nucleus accumbens core versus shell in cue-induced cocaine-seeking behavior. J. Neurosci..

[41] Cleva RM (2011). mGluR5 positive allosteric modulation enhances extinction learning following cocaine self-administration. Behav. Neurosci..

[42] Gass JT, Olive MF (2009). Positive allosteric modulation of mGluR5 receptors facilitates extinction of a cocaine contextual memory. Biol. Psychiatry.

[43] Gass JT (2014). Enhancement of extinction learning attenuates ethanol-seeking behavior and alters plasticity in the prefrontal cortex. J. Neurosci..

[44] Sethna F, Wang H (2014). Pharmacological enhancement of mGluR5 facilitates contextual fear memory extinction. Learn. Mem..

[45] Myers KM, Davis M (2007). Mechanisms of fear extinction. Mol. Psychiatry.

[46] Kinney GG (2005). A novel selective positive allosteric modulator of metabotropic glutamate receptor subtype 5 has in vivo activity and antipsychotic-like effects in rat behavioral models. J. Pharmacol. Exp. Ther..

[47] Paxinos, G. & Watson, C. The Rat Brain in Stereotaxic Coordinates. 6th Edition. Print. Academic Press: San Diego, USA, 2006.

[48] McHugh ML (2013). The chi-square test of independence. Biochem. Med..

[49] McGregor IS, Schrama L, Ambermoon P, Dielenberg RA (2002). Not all “predator odours” are equal: cat odour but not 2,4,5 trimethylthiazoline (TMT; fox odour) elicits specific defensive behaviours in rats. Behav. Brain Res..

[50] Blanchard DC (2003). Failure to produce conditioning with low-dose trimethylthiazoline or cat feces as unconditioned stimuli. Behav. Neurosci..

[51] Wallace KJ, Rosen JB (2000). Predator odor as an unconditioned fear stimulus in rats: elicitation of freezing by trimethylthiazoline, a component of fox feces. Behav. Neurosci..

[52] Marsicano G (2002). The endogenous cannabinoid system controls extinction of aversive memories. Nature.

[53] Sepulveda-Orengo MT, Lopez AV, Soler-Cedeño O, Porter JT (2013). Fear extinction induces mGluR5-mediated synaptic and intrinsic plasticity in infralimbic neurons. J. Neurosci..

[54] Xu J, Zhu Y, Contractor A, Heinemann SF (2009). mGluR5 has a critical role in inhibitory learning. J. Neurosci..

[55] Neumeister A (2013). Elevated brain cannabinoid CB1 receptor availability in post-traumatic stress disorder: a positron emission tomography study. Mol. Psychiatry.

[56] Mantsch JR, Yuferov V, Mathieu-Kia AM, Ho A, Kreek MJ (2004). Effects of extended access to high versus low cocaine doses on self-administration, cocaine-induced reinstatement and brain mRNA levels in rats. Psychopharmacology.

[57] Guillem K, Ahmed SH, Peoples LL (2014). Escalation of cocaine intake and incubation of cocaine seeking are correlated with dissociable neuronal processes in different accumbens subregions. Biol. Psychiatry.

[58] Halbout B, Bernardi RE, Hansson AC, Spanagel R (2014). Incubation of cocaine seeking following brief cocaine experience in mice is enhanced by mGluR1 blockade. J. Neurosci..

[59] Logan CN, LaCrosse AL, Knackstedt LA (2018). Nucleus accumbens GLT-1a overexpression reduces glutamate efflux during reinstatement of cocaine-seeking but is not sufficient to attenuate reinstatement. Neuropharmacology.

[60] Meil WM, See RE (1997). Lesions of the basolateral amygdala abolish the ability of drug associated cues to reinstate responding during withdrawal from self-administered cocaine. Behav. Brain Res..

[61] Stefanik MT, Kalivas PW (2013). Optogenetic dissection of basolateral amygdala projections during cue-induced reinstatement of cocaine seeking. Front. Behav. Neurosci..

[62] Shin LM (2004). Regional cerebral blood flow in the amygdala and medial prefrontal cortex during traumatic imagery in male and female Vietnam veterans with PTSD. Arch. Gen. Psychiatry.

[63] Shin LM (2005). A functional magnetic resonance imaging study of amygdala and medial prefrontal cortex responses to overtly presented fearful faces in posttraumatic stress disorder. Arch. Gen. Psychiatry.

[64] Lissek S (2005). Classical fear conditioning in the anxiety disorders: a meta-analysis. Behav. Res. Ther..

[65] Orr SP (2000). De novo conditioning in trauma-exposed individuals with and without posttraumatic stress disorder. J. Abnorm. Psychol..

[66] Shepard JD, Barron KW, Myers DA (2000). Corticosterone delivery to the amygdala increases corticotropin-releasing factor mRNA in the central amygdaloid nucleus and anxiety-like behavior. Brain Res..

[67] Kozlovsky N, Zohar J, Kaplan Z, Cohen H (2012). Microinfusion of a corticotrophin-releasing hormone receptor 1 antisense oligodeoxynucleotide into the dorsal hippocampus attenuates stress responses at specific times after stress exposure. J. Neuroendocrinol..

[68] Cohen H (2007). Decreased circulatory levels of neuroactive steroids in behaviourally more extremely affected rats subsequent to exposure to a potentially traumatic experience. Int. J. Neuropsychopharmacol..

[69] Kozlovsky N, Matar MA, Kaplan Z, Zohar J, Cohen H (2009). A distinct pattern of intracellular glucocorticoid-related responses is associated with extreme behavioral response to stress in an animal model of post-traumatic stress disorder. Eur. Neuropsychopharmacol..

[70] Windle RJ, Wood SA, Shanks N, Lightman SL, Ingram CD (1998). Ultradian rhythm of basal corticosterone release in the female rat: dynamic interaction with the response to acute stress. Endocrinology.

[71] Morris MC, Compas BE, Garber J (2012). Relations among posttraumatic stress disorder, comorbid major depression, and HPA function: a systematic review and meta-analysis. Clin. Psychol. Rev..

[72] Meewisse ML, Reitsma JB, de Vries GJ, Gersons BPR, Olff M (2007). Cortisol and post-traumatic stress disorder in adults: systematic review and meta-analysis. Br. J. Psychiatry.

[73] Patel S, Roelke CT, Rademacher DJ, Cullinan WE, Hillard CJ (2004). Endocannabinoid signaling negatively modulates stress-induced activation of the hypothalamic-pituitary-adrenal axis. Endocrinology.

[74] Liberzon I, Abelson JL, Flagel SB, Raz J, Young EA (1999). Neuroendocrine and psychophysiologic responses in PTSD: a symptom provocation study. Neuropsychopharmacology.

[75] Elzinga BM, Schmahl CG, Vermetten E, van Dyck R, Bremner JD (2003). Higher cortisol levels following exposure to traumatic reminders in abuse-related PTSD. Neuropsychopharmacology.

[76] Brodnik ZD (2017). Susceptibility to traumatic stress sensitizes the dopaminergic response to cocaine and increases motivation for cocaine. Neuropharmacology.

[77] Meyer EM, Long V, Fanselow MS, Spigelman I (2013). Stress increases voluntary alcohol intake, but does not alter established drinking habits in a rat model of posttraumatic stress disorder. Alcohol Clin. Exp. Res..

[78] Pizzimenti CL, Navis TM, Lattal KM (2017). Persistent effects of acute stress on fear and drug-seeking in a novel model of the comorbidity between post-traumatic stress disorder and addiction. Learn. Mem..

[79] Ferland CL, Reichel CM, McGinty JF (2016). Effects of oxytocin on methamphetamine-seeking exacerbated by predator odor pre-exposure in rats. Psychopharmacology.

